# Improved cold tolerance in *Elymus nutans* by exogenous application of melatonin may involve ABA-dependent and ABA-independent pathways

**DOI:** 10.1038/srep39865

**Published:** 2017-01-03

**Authors:** Juanjuan Fu, Ye Wu, Yanjun Miao, Yamei Xu, Enhua Zhao, Jin Wang, Huaien Sun, Qian Liu, Yongwei Xue, Yuefei Xu, Tianming Hu

**Affiliations:** 1 Department of grassland science, College of Animal Science and Technology, Northwest A&F University, Yangling, Shaanxi, 712100, China; 2 College of Plant Science, Tibet Agriculture and Animal Husbandry College, Linzhi, Tibet, 860000, China; 3 Department of grassland ecology, College of Desertification Prevention Engineering, Ningxia Technical College of Wine and Desertification Prevention,Yongning, Yinchuan, 750001, China

## Abstract

Melatonin is an important secondary messenger that plays a central role in plant growth, as well as abiotic and biotic stress tolerance. However, the underlying physiological and molecular mechanisms of melatonin-mediated cold tolerance, especially interactions between melatonin and other key molecules in the plant stress response, remain unknown. Here, the interrelation between melatonin and abscisic acid (ABA) was investigated in two genotypes of *Elymus nutans* Griseb., the cold-tolerant Damxung (DX) and the cold-sensitive Gannan (GN) under cold stress. Pre-treatment with exogenous melatonin or ABA alleviated oxidative injury via scavenging ROS, while enhancing both antioxidant enzyme activities and non-enzymatic antioxidant contents. Treatment of fluridone, an ABA biosynthesis inhibitor caused membrane lipid peroxidation and lowered melatonin-induced antioxidant defense responses. It is worth noting that cold stress significantly induced both endogenous melatonin and ABA levels in both genotypes. Application of melatonin increased ABA production, while fluridone significantly suppressed melatonin-induced ABA accumulation. ABA and fluridone pre-treatments failed to affect the endogenous melatonin concentration. Moreover, exogenous melatonin up-regulated the expression of cold-responsive genes in an ABA-independent manner. These results indicate that both ABA-dependent and ABA-independent pathways may contribute to melatonin-induced cold tolerance in *E. nutans*.

Cold stress presents one of the major limitations for plant growth and yield worldwide, especially in areas of high altitude due to its negative effects on plant physiology, biochemistry, and molecular biology^1^. Changes in membrane fluidity and composition under cold exposure trigger the accumulation of various osmoprotectants, thus alleviating oxidative damage^2^,^3^. At molecular level, low but non-freezing temperatures have been reported to induce rapid expression of transcription factors and cold-regulated genes, resulting in an enhanced freezing tolerance^4^. This complex response is comprised of two major pathways and which one will be utilized depends on the involvement of abscisic acid (ABA)^5^. One of the known major ABA-independent cold-signaling pathways is the ICE (inducer of CBF expression)–CBF (C-repeat binding factor)–COR (cold regulated genes) transcriptional cascade^6^. In cereals, *CBF14* was found to have maximal effect on freezing tolerance^7^,^8^,^9^. One of the first detectable gene expressions induced by low temperatures was the rapid and controlled induction of *CBF* genes, increasing simultaneously cold tolerance^7^. The *CBF* response is furthermore followed by the expression of the *COR* gene^10^. In Triticeae, *COR* expression levels are correlated with freezing tolerance: genotypes with better freezing tolerance accumulate *COR* gene transcripts in higher amounts compared to genotypes with lower freezing tolerance^11^. The second pathway participating in the cold acclimation process is ABA-dependent and is induced via dehydration instead of the lowered temperature itself. This response is slow and it includes bZIP transcription factors known as ABA Responsive Element Binding Protein/Factors (AREB/ABF)^12^. Both pathways are not independent, but are linked via complex interrelations^13^,^14^.

Hormone treatment has been utilized as an approach to alleviate various abiotic and biotic stresses in plants^15^. Melatonin (N-acetyl-5-methoxytryptamine) is an important animal hormone that has been reported to be involved in multiple biological processes^16^. Although its function as a hormone has been well established in animals, functional knowledge in higher plant is still very limited. In recent years, melatonin has been found to be a ubiquitous modulator in multiple plant developmental processes, including flowering, promotion of photosynthesis, preservation of chlorophyll^17^, stimulation and regeneration of root system architecture^18^, delayed senescence of leaves^19^, and alleviation of oxidative damage induced by reactive oxygen species (ROS) and reactive nitrogen species (RNS)^17^,^20^. Moreover, melatonin is involved in the regulation of various abiotic stresses, such as cold^21^,^22^,^23^, salinity^24^, heavy metal^25^, herbicides^26^, and UV radiation^27^. The mechanisms of melatonin-mediated stress tolerance involve the activation of antioxidants biosynthesis and activities of antioxidant enzymes, as well as the direct scavenging of ROS following plant exposure to harsh environments^28^,^29^,^30^. Despite melatonin positively mediating plant responses to cold stress (e.g. via promoting seed germination in cucumber^28^, mitigating oxidative damage in maize seedlings^23^, and up-regulating the expression of cold-induced transcriptional activators including *CBFs* and zinc finger of *Arabidopsis thaliana 6* (ZAT6)^30^,^31^), the exact mechanism enabling these responses remain largely unknown.

The plant hormone ABA acts as a stress signal in plants and plays an important role in modulating plant response to various biotic and abiotic stresses, including cold stress^32^. A previous study showed that exogenous ABA application before the onset of cold stress improved cold resistance of plants^33^. ABA can improve the antioxidant defense system, thus protecting plant cells from damage caused by over-accumulated ROS^34^. Accumulating evidence has shown that ABA interacts with other important signaling molecules, such as nitric oxide (NO), hydrogen peroxide (H_2_O_2_), and calcium (Ca^2+^), thus participating in the regulation of cold-tolerance responses^35^,^36^. However, the interrelation between melatonin and ABA in the acquisition of cold tolerance remains unclear.

To improve our understanding of melatonin function and its potential interrelation with ABA in plants exposed to cold conditions, two genotypes of *Elymus nutans* Griseb., the cold-tolerant Damxung (DX) and the cold-sensitive Gannan (GN) were studied. *E. nutans* is a perennial Triticeae cool-season grass, with a distribution in the northern, northwestern, and southwestern regions of China. It is especially prevalent on the alpine meadow of the Qinghai-Tibetan Plateau where temperatures greatly fluctuate^37^. Thus, these grasses have evolved specific physiological mechanisms to adapt to changing environmental conditions. A detailed analysis of the cold adaptation of *E. nutans* will expand our understanding of cold tolerance in plants in general. In this study, the endogenous melatonin levels and cold-responsive genes were examined and quantified in both genotypes when exposed to cold stress. Our data revealed that endogenously produced melatonin and expressions of *EnCBFs* and *EnCOR14a* genes were significantly increased due to cold stress in both genotypes. Based on pharmacological and biochemical analyses, the present study suggests that melatonin-induced cold tolerance in *E. nutans* works via both ABA-dependent and ABA-independent pathways.

## Results

### Exogenous melatonin and ABA alleviates cold stress–induced growth inhibition and cell membranedamage

**Figure 1:**
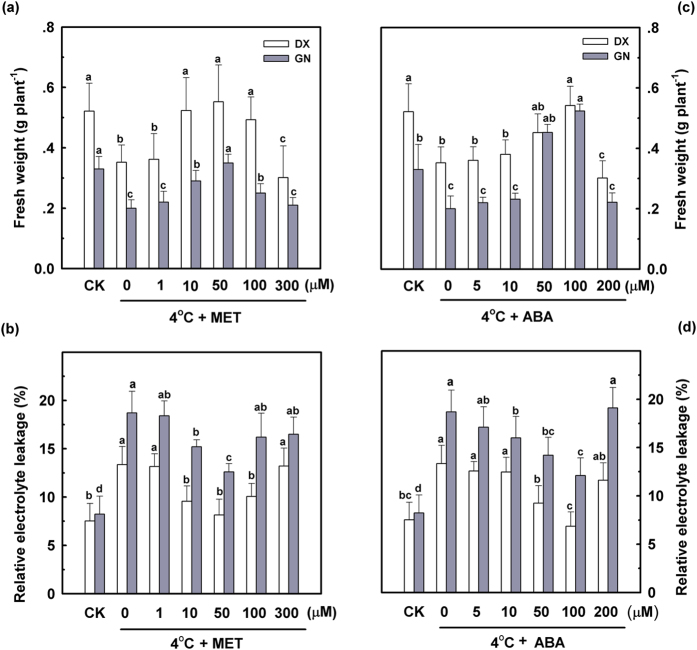
Effects of different melatonin and ABA concentrations on fresh weight (**a,c**) and electrolyte leakage (**b,d**) in DX and GN seedlings under cold stress (4 °C). Plants treated with distilled water under the normal conditions (25 °C) served as controls. Each value represents the mean of three replicates ± SD, and different letters above the bars indicate significant differences at *P* < 0.05 among different treatments in the same genotype according to Duncan’s multiple range test.

Cold stress resulted in significant growth suppression in two *E. nutans* seedlings, with fresh weights decreasing by 39.4% (GN) and 32.1% (DX) compared to the control during the 120 h of cold treatment (Fig. 1a,c). The relative electrolyte leakage level was increased (*P* < 0.05) in both genotypes under cold stress, and values were lower in DX than in GN (Fig. 1b,d).

To determine the appropriate level of melatonin for plant growth, fresh weight of the two *E. nutans* genotypes were compared following cultivation on various melatonin levels (0–300 μM). Our results revealed that the best growth and development could be obtained with melatonin concentrations ranging from 10 to 50 μM after 120 h of cold stress, whereas higher melatonin doses (300 μM) had no protective effect on (*P* < 0.05) plant growth (Fig. 1a). Exogenous melatonin treatment had no significant effect on shoot length and fresh weight under normal conditions (Supplementary Table S1). Similarly, melatonin affected electrolyte leakage in a dosage-dependent manner when plants were subjected to cold stress. While melatonin concentrations of 10–100 μM reduced cold stress-induced electrolyte leakage, reaching an optimal dose at 50 μM melatonin treatment, higher concentrations of melatonin (300 μM) resulted in no protective effect on membranes (Fig. 1b). In summary, 50 μM melatonin was most effective to improve the resistance to cold-induced membrane damage for both tested genotypes.

Pre-treatment with exogenous ABA was performed at concentrations of 0, 5, 10, 50, 100, and 200 μM to determine the concentration that leads to the most significant effect on cold stress tolerance. The results show that ABA concentrations from 50 to 100 μM alleviated the loss of fresh weight as well as the electrolyte leakage level, with 100 μM being most effective in the leaves of DX and GN under cold stress (Fig. 1c,d). Since 50 μM melatonin and 100 μM ABA appeared to be the most effective in enhancing cold tolerance in *E. nutans*, these concentrations were used in all subsequent experiments.

### ABA is involved in melatonin-induced reduction in oxidative damage

**Figure 2:**
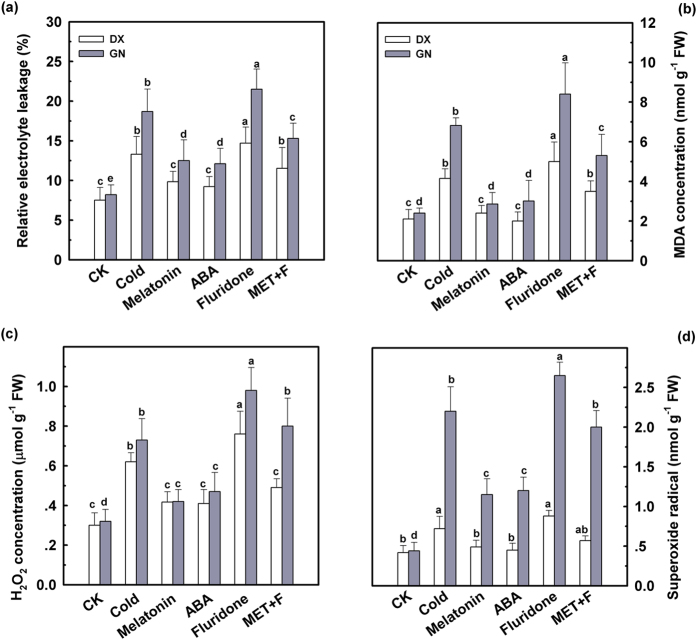
Effect of melatonin and ABA on electrolyte leakage (**a**), MDA content (**b**), H_2_O_2_ (**c**) and superoxide radical (**d**) accumulation in leaves of DX and GN under cold stress. The 21-day-old seedlings were pretreated with distilled water, 50 μM melatonin, 100 μM ABA, 50 μM fluridone, and 50 μM melatonin + 50 μM fluridone (MET + F) for 7 days, respectively, and then exposed to cold stress (4 °C) for 120 h. Plants treated with distilled water under the normal conditions (25 °C) served as controls. Each value represents the mean of three replicates ± SD, and different letters above the bars indicate significant differences at *P* < 0.05 among different treatments in the same genotype according to Duncan’s multiple range test.

To investigate the role of ABA in melatonin alleviated oxidative damage caused by cold stress, an inhibitor of endogenous ABA biosynthesis was used (fluridone). The electrolyte leakage, MDA concentrations, and ROS levels for each *E. nutans* genotypes were increased (*P* < 0.05) after 120 h of cold stress and values were lower in the cold-tolerant DX than in the cold-sensitive GN (Fig. 2a–d). However, exogenous application of 50 μM melatonin or 100 μM ABA to the roots via irrigation solution alleviated (*P* < 0.05) this response over the course of the stress period. The beneficial effects of exogenous melatonin and ABA were more profound for the cold-sensitive genotype than for cold-tolerant plants, as indicated by melatonin-induced declines in electrolyte leakage, MDA concentration, H_2_O_2_ accumulation, and superoxide radical production (by 26%, 42.2%, 32.7%, and 37.1% (tolerant) vs 33.2%, 58.1%, 42.5%, and 47.7% (sensitive), respectively). Pre-treatment with fluridone under simultaneous presence of melatonin evidently increased electrolyte leakage, MDA concentration, and ROS levels. However, these treatments alone failed to affect membrane lipid peroxide levels and ROS concentrations in control leaves (Supplementary Table S2). These results revealed ABA involvement in exogenous melatonin-induced reduction in membrane lipid peroxide levels and ROS concentrations.

### ABA is required for exogenous melatonin-induced antioxidant defense

**Figure 3:**
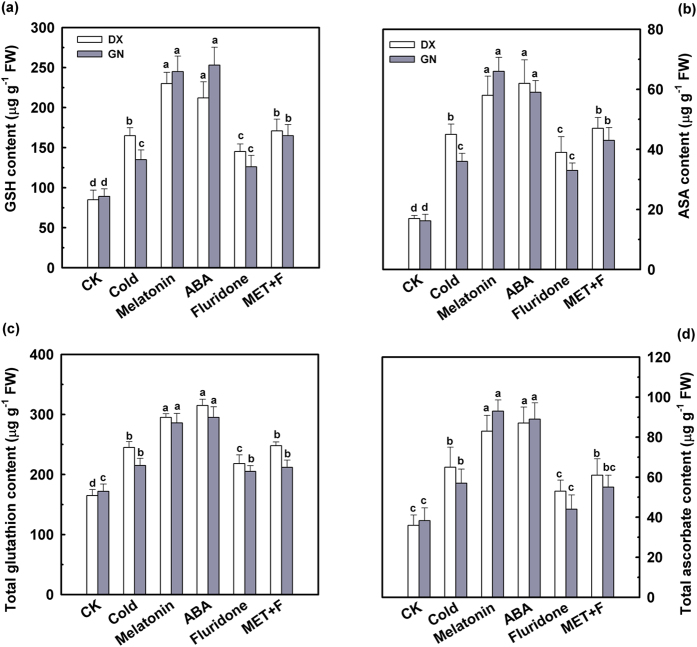
Effect of melatonin and ABA on contents of non- enzymatic antioxidants GSH (**a**), AsA (**b**), Total glutathione (**c**) and ascorbate (**d**) in leaves of DX and GN under cold stress. The 21-day-old seedlings were pretreated with distilled water, 50 μM melatonin, 100 μM ABA, 50 μM fluridone, and 50 μM melatonin + 50 μM fluridone (MET + F) for 7 days, respectively, and then exposed to cold stress (4 °C) for 120 h. Plants treated with distilled water under the normal conditions (25 °C) served as controls. Each value represents the mean of three replicates ± SD, and different letters above the bars indicate significant differences at *P* < 0.05 among different treatments in the same genotype according to Duncan’s multiple range test.

**Figure 4:**
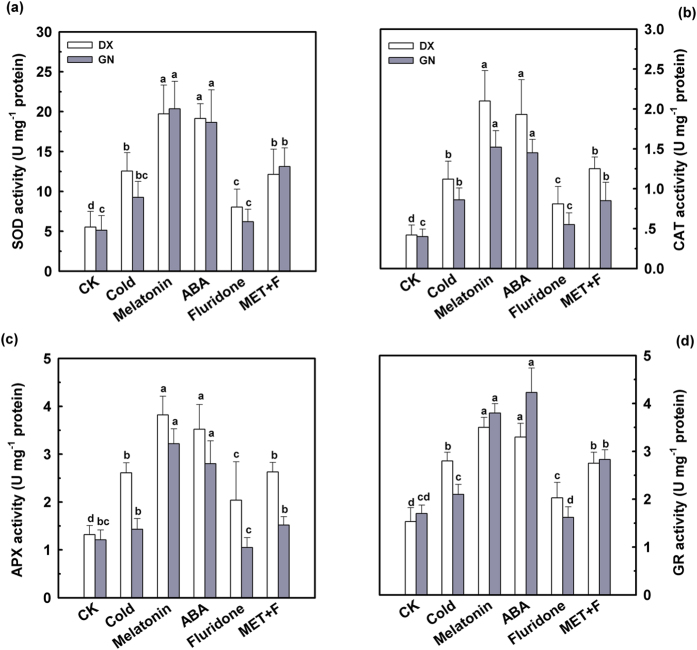
Effect of melatonin and ABA on the activities of antioxidant enzyme SOD (**a**), CAT (**b**), APX (**c**) and GR (**d**) in leaves of DX and GN under cold stress. The 21-day-old seedlings were pretreated with distilled water, 50 μM melatonin, 100 μM ABA, 50 μM fluridone, and 50 μM melatonin + 50 μM fluridone (MET + F) for 7 days, respectively, and then exposed to cold stress (4 °C) for 120 h. Plants treated with distilled water under the normal conditions (25 °C) served as controls. Each value represents the mean ± SD of three repeats, and different letters above the bars indicate significant differences at *P* < 0.05 among different treatments in the same genotype according to Duncan’s multiple range test.

Cold stress caused an increase in non-enzymatic antioxidant concentrations and in the activities of antioxidant enzymes in leaves from all sampled plants. Values were further increased via application of melatonin and ABA. An exogenously applied 50 μM of fluridone led to a decline in concentrations of GSH, AsA, total glutathione, and total ascorbate after 120 h of cold stress. Combining melatonin and fluridone declined (*P* < 0.05) the concentrations of these antioxidants (Fig. 3a–d). Activities of SOD, CAT, APX, and GR (Fig. 4a–d) increased due to melatonin exposure under cold stress, especially in GN leaves. However, pre-treatment with fluridone alone did not affect the concentrations of non-enzymatic antioxidants or the activities of antioxidant enzymes in the control leaves (Supplementary TablesS3 and S4). These results suggest that ABA is required for melatonin-induced stimulation of antioxidant defense and alleviating oxidative damage in cold-stressed *E. nutans* leaves.

### Endogenous melatonin and ABA concentrations in response to cold stress

**Figure 5:**
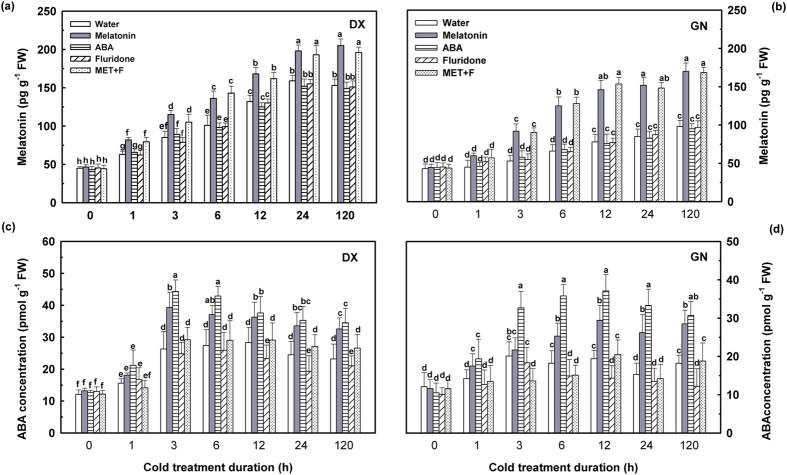
Endogenous melatonin (**a**) and ABA (**b**) concentration in leaves of DX and GN under cold stress. The 21-day-old seedlings were pretreated with distilled water, 50 μM melatonin, 100 μM ABA, 50 μM fluridone, and 50 μM melatonin + 50 μM fluridone (MET + F) for 7 days, respectively, and then exposed to cold stress (4 °C). Samples were taken at 0, 1, 3, 6, 12, 24, and 120 h of cold treatment. Untreated controls were taken at time zero after the experiment began. Each value represents the mean of three replicates ± SD, and different letters above the bars indicate significant differences at *P* < 0.05 among different time points in the same genotype according to Duncan’s multiple range test.

To reveal the relationship between cold stress and melatonin concentration, the endogenous melatonin levels in leaves of DX and GN were quantified after exposure to cold stress (4 °C) for 0, 1, 3, 6, 12, 24, and 120 h. Endogenous melatonin contents of both genotypes were approximately 45 pg g^−1^ FW before transfer to cold conditions. When exposed to cold stress (4 °C), the endogenous melatonin content in both genotypes continuously increased compared to plants cultured in control conditions within 120 h cold stress (Fig. 5a,b). Melatonin-treated plants showed a further increase in endogenous melatonin content compared to non-treated plants during the whole cold stress period. This was even more marked in sensitive species, demonstrating that those plants could not produce melatonin as efficiently as plants with improved cold tolerance. This significant induction of the endogenous melatonin concentration via cold stress indicated a direct involvement of melatonin in the *E. Nutans* response to cold stress. Accordingly, the endogenous ABA levels in both species immediately increased when subjected to 1 h of cold stress, reached their maximal level at 3 h, and remained at similar levels from 6 h to 120 h. Notably, increased endogenous ABA levels were observed in DX compared to GN after 3 h of cold stress, lasting until the experiment ended. A further increase of endogenous ABA concentration was observed after exogenous melatonin treatment, while the increases were blocked (*P* < 0.05) by pre-treatment with the ABA biosynthesis inhibitor fluridone in both plants during the cold treatment period (Fig. 5c,d). The inhibitory effects were more pronounced for cold-sensitive GN than for the cold-tolerant DX. Fluridone treatment had no significant effect on ABA concentration in both *E. nutans* genotypes grown in normal conditions (Supplementary Table S5). Pre-treatment with exogenous melatonin and melatonin + fluridone (MET + F) increased (*P* < 0.05) endogenous melatonin contents in leaves of DX and GN throughout the treatment. However, application of ABA or fluridone alone failed to affect the endogenous melatonin production in both genotypes compared to non-treated plants under cold stress. This indicates that melatonin-induced cold signaling pathway might be ABA-independent.

### Effect of melatonin and ABA on cold-regulated genes

**Figure 6:**
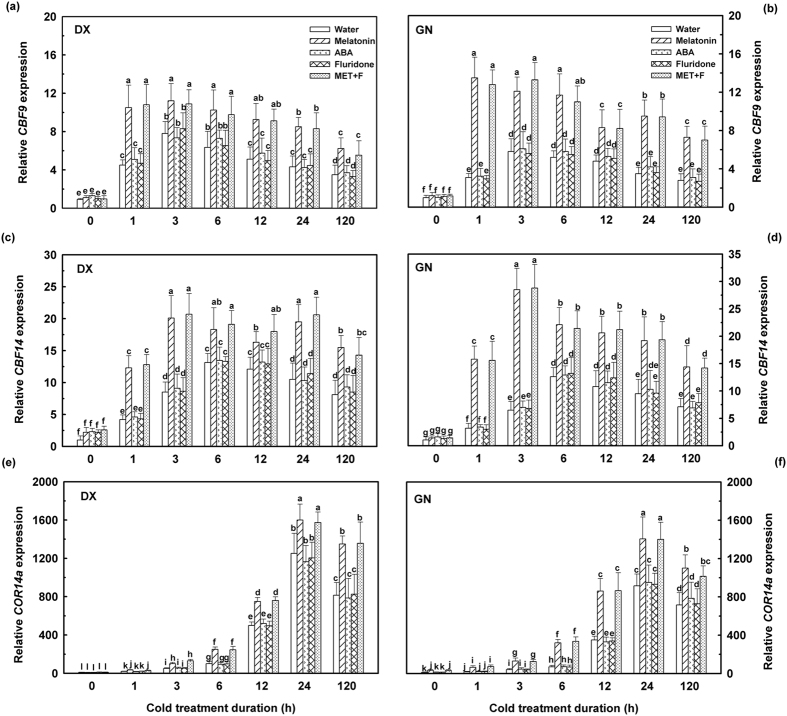
Expression of cold-related genes in leaves of DX and GN under cold stress. The 21-day-old seedlings were pretreated with distilled water, 50 μM melatonin, 100 μM ABA, 50 μM fluridone, and 50 μM melatonin + 50 μM fluridone (MET + F) for 7 days, respectively, and then exposed to cold stress (4 °C). Samples were taken at 0, 1, 3, 6, 12, 24, and 120 h of cold treatment. Untreated controls were taken at time zero after the experiment began. Each value represents the mean of three replicates ± SD, and different letters above the bars indicate significant differences at *P* < 0.05 among different time points in the same genotype according to Duncan’s multiple range test.

Cold stress has been reported to induce the expression of the CBF family of transcription fators, which in turn activate a set of downstream *COR* genes protecting plants from cold-induced injury^4^,^5^. According to previous transcriptome data, the levels of *EnCBF9, EnCBF14*, and *EnCOR14a* were significantly induced due to cold treatment in both *E. nutans* genotypes (Supplementary Table S6). As shown in Fig. 6, the expressions of *EnCBF9* and *EnCBF14* were immediately induced in both genotypes exposed to cold treatment at 1 h and 3 h, and reached a maximal value at 3 h and 6 h, followed by a decline to levels still well above those prior to cold exposure. Compared to non-melatonin-treated plants under cold stress, both genotypes treated with exogenous melatonin had higher (*P* < 0.05) levels of *EnCBF9* and *EnCBF14* transcripts during cold stress. The expression levels of *EnCBF9* immediately increased within 1 h of exposure to cold stress and melatonin treatment, and decreased until 12 h, while increasing at 24 h. After 120 h of cold treatment, the expression level of *EnCBF9* declined in the melatonin-treated plants, but significantly increased (*P* < 0.05) compared to non-melatonin-treated plants under cold stress. The transcript of *EnCBF14* genes in GN genotypes immediately increased within 1 h of exposure to cold stress and melatonin treatment, peaked at 3 h, and subsequently declined until 120 h of cold treatment. Pre-treatment with ABA and fluridone had no significant inhibitory effect on the expression of the two *EnCBFs* in cold treated *E. nutans*.

The transcript levels of *EnCOR14a* increased (*P* < 0.05) in both genotypes following 6 h of cold stress and continued to increase for up to 24 h, before decreasing thereafter (Fig. 6e,f). The relative normalized expression of *EnCOR14a* was significantly higher in melatonin-treated plants compared to untreated plants during the 120 h of cold stress. A 2.5-fold up-regulation in DX and a 4.6-fold up-regulation in GN were observed in melatonin treated plants after 6 h of cold treatment. Combination of melatonin and fluridone treatment did not lead to an inhibitory effect in the expression of *COR14a* compared with melatonin and cold stress treatments. Based on our results, we propose that one of the mechanisms of melatonin may be to protect plant cells from cold injury by regulating cold responsive genes, including CBF transcription factors and cold-responsive genes.

## Discussion

Cold stress is one of the most important abiotic stresses limiting plant growth and causes severe dysfunctions at cellular level. The primary place of cold injury is the cell membrane system^38^. In this study, cold stress resulted in measureable growth restriction and severe oxidative damage in the cold-sensitive GN genotype indicated by increases in electrolyte leakage level, MDA content, and accumulations of O_2_^•−^ and H_2_O_2_. Exogenously applied melatonin alleviated this growth limitation and protected membrane structures against peroxidation during cold stress (Figs 1 and 2). A decline in relative electrolyte leakage and MDA concentration confirmed the protective effect of melatonin in cold stress-induced membrane damage. Melatonin and several of its metabolites are known endogenous free radical scavengers and broad-spectrum antioxidants and have been suggested to directly scavenge ROS^25^,^28^,^30^. Our results revealed that the generation of H_2_O_2_ and O_2_^•−^ was inhibited by exogenous melatonin under cold stress. Application of melatonin led to a decline in ROS production, which might be due to direct enhancement of GSH and AsA concentrations as well as CAT, SOD, APX, and GR activities under cold stress (Figs 3 and 4). Pre-treatment with melatonin had a more profound effect on the cold-sensitive genotype compared to the cold-tolerant genotype. These results are consistent with previous study, reporting a primarily antioxidant function of melatonin in plants against abiotic and biotic stresses^12^.

Ample evidence supports the regulatory role of ABA in conferring tolerance to environmental stresses^33^,^39^. Here, we found that exogenous pre-treatment with ABA efficiently ameliorated cold-induced oxidative damage, characterized by decreased electrolyte leakage, reduced MDA content, and decreased ROS accumulation (Fig. 2). In addition, pre-treatment with ABA enhanced GSH, AsA, total glutathione, and total ascorbate concentrations, as well as SOD, CAT, APX, and GR activities in both genotypes under cold stress. Similar conclusions were observed in pepper under ABA and low temperature conditions^40^. It has been well documented that ABA interacted with the important signaling molecules NO, H_2_O_2_, and Ca^2+^, participating in the regulation of responses to cold tolerance. However, it is not clear whether ABA is involved in melatonin-induced cold-tolerance in *E. nutans*. To further investigate the roles of ABA signaling in the response to cold stress and exogenous melatonin treatment, fluridone, an inhibitor of endogenous ABA biosynthesis, was used. When exposed to cold stress, pre-treatments with fluridone in the presence of melatonin inhibited the positive effect of melatonin for cell membranes as indicated by increases in electrolyte leakage, MDA concentration, and ROS levels, and a decline in antioxidant enzymes activities (Figs 3 and 4). These observations suggest that ABA is likely participating in melatonin-induced antioxidant defense under cold stress.

To further investigate the role of melatonin and ABA in the antioxidant defense induced by cold stress, endogenous melatonin and ABA concentrations were assayed. Exposure of *E. nutans* seedlings to 0–120 h of cold stress gradually and substantially increased endogenous melatonin and ABA concentrations (Fig. 5). However, such increases in endogenous melatonin were unable to ameliorate oxidative stress, which is possibly due to insufficient ROS scavenging by melatonin in parallel with cold-induced ROS generation, especially in cold-sensitive genotype. The higher accumulation of melatonin and ABA in DX compared to GN leaves might contribute to the increased cold tolerance of DX. In agreement with our finding, melatonin-deficient tomatoes were less protected against abiotic stress when compared to the wild type tomato^41^, whereas melatonin-rich plants had an improved ability to withstand such challenges^29^. Accumulation of endogenous melatonin has been suggested to be an adaptive response in plants under cold stress^42^. Treatment with exogenous melatonin significantly increased endogenous melatonin content during cold treatment. As expected, application of melatonin had a more profound effect on cold-sensitive GN than on cold-tolerant DX genotype. It is possible that a mechanism of exogenous melatonin alleviating oxidative injury induced by cold stress, may activate endogenous melatonin synthesis, which in turn acts as an antioxidant and further mediates other defensive pathways for subsequent environmental adaptation. In the present study, an important finding was observed related to melatonin and antioxidant defense induced by cold stress. Melatonin application rapidly enhanced ABA levels in the leaves of both *E. nutans* genotypes, while ABA as well as ABA inhibitor applications had no significant effect on endogenous melatonin production (Fig. 5). These observations demonstrated that ABA might act as a downstream signal of melatonin in the antioxidant defense responses of plants.

Recent studies have reported that melatonin plays central roles in the regulation of gene expression and antioxidant activities^30^. Several important cold-related genes were selected. The present result indicated that exogenous melatonin up-regulated components of the cold-stress signaling pathways following specific time intervals of cold exposure. Transcripts of *EnCBF9* and *EnCBF14* accumulated immediately after 1 and 3 h of cold treatment. The induction of *CBFs* in plants up-regulated the expression of COR genes such as *COR14b*, leading to increased freezing tolerance^10^. Cold stress induced higher *EnCOR14a* expression in the cold-tolerant genotypes DX compared to the cold-sensitive genotype GN (Fig. 6), thus supporting the findings of Ndong *et al*.^11^ who pointed out that group 3 late embryogenesis abundant proteins were induced in cold-treated wheat and rye plants. The increased expression of *EnCBF9, EnCBF14*, and *EnCOR14a* was induced by melatonin treatment, revealed by a comparison with untreated plants under cold stress. These observations indicate that melatonin might serve as a second messenger, activating downstream cold-responsive genes including *EnCBF9, EnCBF14*, and *EnCOR14a*, and thus stimulating biosynthesis of cold-protecting compounds. Consequently, this contributes to the enhancement of cold tolerance in plants treated with exogenous melatonin and subjected to cold stress^23^,^31^. Previous studies reported that both cold treatment and exogenous ABA treatment induce the expression of *CBF1* and several *COR* genes in *A. thaliana* plants^43^,^44^. In contrast, our study revealed that application of exogenous ABA and fluridone failed to further change expressions of *EnCBFs* and *EnCOR14a* genes under cold stress. This however, is in accordance with the findings of Skinner *et al*.^45^ who found that ABA-treated barley under cold stress did not affect *CBF* gene expression. The results suggested that activation of *CBF* and *COR* gene expression can regulated through both ABA-dependent and ABA-independent pathways^5^,^46^.

## Conclusions

We found that both exogenous melatonin and ABA have the ability to alleviate cold stress induced oxidative damage. Exogenous melatonin improved cold tolerance via induction of endogenous melatonin production, which might serve as a second messenger activating downstream cold-responsive genes such as *EnCBF9, EnCBF14*, and *EnCOR14a*, thus stimulating antioxidant defense systems alleviating ROS accumulation-induced oxidative damage. Further investigations revealed that this melatonin-induced antioxidant defense may function via ABA-dependent and ABA-independent signaling pathways. However, the molecular network that operates during cold stress and that is mediated by melatonin remains to be determined. Further studies are necessary to elucidate the interrelation between melatonin and other signaling molecules in response to cold stress.

## Materials and Methods

### Plant materials and experimental design

*Elymus nutans* Griseb. seeds were obtained from two sources: seeds of Damxung (DX) were collected in September 2015, from wild plants growing in Damxung County (30°28.535′N, 91°06.246′E, altitude 4678 m), located in the middle of Tibet, China. And Gannan (GN) seeds were obtained from Lanzhou Xinglong Grass Industry Technology Service CO. Ltd., China in September 2015. The seeds were cleaned and stored at 4 °C in paper bags until the start of the experiments.

DX and GN seeds were surface-sterilized in 0.1% (w/v) sodium hypochlorite, and germinated on moistened filter paper for 7 days at 25 °C. Morphologically uniform seedlings were transferred to plates using a 1: 1 (v/v) mixture of vermiculite and sand as solid support. Plants were germinated and grown in a growth chamber at a day/night temperature 25/25 °C, a relative humidity of 70/60%, a day/night regime of 14/10 h and a photosynthetic photon flux density (PPFD) of 300 μmol m^−2^ s^−1^. Light was provided by a fluorescent lamp (Philips Electronics N. V. Holland, Nanjing, China). To investigate the role of exogenous melatonin and ABA in plant physiological responses and cold stress resistance, 21-day-old *E. nutans* seedlings were irrigated with water or with different concentrations of melatonin (0, 1, 10, 50, 100, 300 μM), ABA (0, 5, 10, 50, 100, 200 μM), and 50 μMfluridone (ABA biosynthesis inhibitor) for 7 days, respectively^31^. After 7 days of pretreatment, the 28-day-old plants were subjected to cold stress (4 °C) for 120 h. Samples were taken at 0, 1, 3, 6, 12, 24, and 120 h of cold treatment, immediately flash frozen in liquid nitrogen and stored at −80 °C until analyzed. After 120 h of cold treatment, fresh weight of shoots, cell membrane response, ROS accumulation, non-enzymatic antioxidants concentration and related antioxidant enzyme activities were measured. For expression of cold-related genes, endogenous melatonin, and ABA content were quantification at different time intervals of cold stress. Untreated controls were taken at time zero after the experiment began. All assessments were conducted in three biological replicates. Three replicates from independent plates (each consisting of 15 plants) were harvested for each treatment.

### Determination of plant growth characters

Three healthy seedlings were randomly chosen from each group after 120 h of cold stress. The shoots of the seedlings were cut at the growth medium line and their fresh weights were recorded.

### Assay of electrolyte leakage and malondialdehyde content

Electrolyte leakage was determined according to the method of Song *et al*.^47^ with some modifications. The fresh leaves (0.5 g) were washed in deionized water and placed in Petri dishes with 5 ml deionized water at 25 °C for 2 h. After the incubation, the conductivity was measured. Then, the samples were boiled for 20 min and conductivity was read again. The electrolyte leakage was expressed as percent.

Malondialdehyde (MDA) content was extracted using chilled thiobarbituric acid (TBA) reagent according to the method of Dhindsa *et al*.^48^. The absorbance of the supernatant was measured at 450, 532, and 600 nm.

### Quantification of ROS Accumulation

The concentration of H_2_O_2_ was measured by monitoring the absorbance of titanium-peroxide complex at 415 nm according to the method described by Shi *et al*.^49^.

Superoxide radical production rate was determined by the plant O_2_^•−^ ELISA Kit (DG, Beijing, China) based on antibody-antigen-enzyme-antibody complex following the manufacturer’s instruction.

### Measurement of Non-enzymatic Antioxidant Concentrations and Antioxidant Enzyme Activities

A leaf sample (0.4 g) was ground with a mortar and pestle in 2 ml of 0.5 M EDTA solution containing 3% trichloroacetic acid and centrifuged at 15,000 × *g* for 10 min at 4 °C. The supernatant was used for assays of the levels of ascorbic acid (AsA) and glutathione (GSH). The amount of GSH was evaluated following the method of Anderson^50^ and was expressed as μg g^−1^ FW. The amount of AsA was estimated using the method of Foyer and Halliwei^51^ and was expressed as μg g^−1^ FW.

The leaves (0.5 g) were homogenized with a mortar and pestle at 4 °C in 5 ml 50 mM phosphate buffer (pH 7.8) containing 1 mM EDTA and 2% PVP. Homogenate was centrifuged at 12,000 × *g* for 20 min at 4 °C and the supernatant was used for enzyme activity assays. Protein content in the supernatant was determined according to Bradford^52^.

The assay for ascorbate peroxidase (APX) activity was measured in a reaction mixture of 3 ml containing 100 mM phosphate (pH 7), 0.1 mM EDTA-Na_2_, 0.3 mM ascorbic acid, 0.06 mM H_2_O_2_ and 100 μl enzyme extract. Change in absorption was observed at 290 nm 30 s after addition of H_2_O_2_^53^. One unit of APX forms 1 μM of ascorbate oxidized per minute under assay conditions. The activity of catalase (CAT) was measured by following the consumption of H_2_O_2_ at 240 nm according to Cakmak and Marschner^54^. The decrease in the absorption was followed for 3 min and a breakdown of 1.0 μM H_2_O_2_ ml^−1^min^−1^ was defined as 1 Unit of CAT activity. Glutathione reductase (GR) activity was measured by following the decrease in absorbance at 340 nm due to NADPH oxidation. The reaction mixture contained tissue extract, 1 mM EDTA, 0.5 mM GSSG, 0.15 mM NADPH and 50 mM Tris–HCl buffer (pH 7.5) and 3 mM MgCl_2_^55^. The reaction was started by adding NADPH. Activity of superoxide dismutase (SOD) was determined according to Beauchamp and Fridovich^56^ by following the photo-reduction of nitroblue tetrazolium (NBT) at 560 nm.

### Quantification of Endogenous Melatonin by Enzyme-linked Immunosorbent Assay

Melatonin was extracted using the acetone–methanol method as previously described^57^. The supernatant was transfered to a new centrifuge tube containing 0.5 ml of 1% trichloric acid for protein precipitation. After centrifugation at 12,000 × *g* for 10 min at 4 °C, the extract was used for quantification of melatonin using the Plant Melatonin ELISA Kit (RD, USA).

### Determination of ABA Content

The quantitative determination of ABA in leaves of DX and GN plants were carried out using enzyme-linked immunosorbent assay (RD, USA). Extraction and purification prior to immunoassay have been described by Zhang *et al*.^58^.

### Extraction of Total RNA and Quantitative Real-time PCR (qRT-PCR) Analyses

Total RNA was isolated from leaves using RNAiso Reagent (TaKaRa, Dalian, China). Gel electrophoresis was performed and absorbance measured at 260 and 280 nm to ensure RNA integrity. RNA was reverse-transcribed with PrimeScript RT reagent Kit with gDNA Eraser (Takara, Dalian, China). Synthesized cDNA was subjected to Polymerase chain reaction (PCR) for 40 cycles using primers as described in Table 1. The PCR primers were designed using Primer Premier 5 (Version 5.0 for Windows and Power Macintosh, Palo Alto, CA). Each gene was obtained from our previous transcriptome data of *E. nutans* (accession No: SRP074469). The PCR conditions consisted of denaturation at 95 °C for 3 min, followed by 40 cycles of denaturation at 95 °C for 30 s, annealing at 60 °C for 30 s, and extension at 72 °C for 30 s. The relative expression levels of target gene were calculated with formula 2^−ΔΔCT^ method^59^.

### Statistical Analysis

Each experiment was repeated three times. All values were expressed as means ± SD. The data were analyzed via one-way ANOVA using SPSS-17 statistical software (SPSS Inc., Chicago, IL, USA), followed by Duncan’s tests. A *p*-value of <0.05 indicated a significant difference.

## Additional Information

**How to cite this article**: Fu, J. *et al*. Improved cold tolerance in *Elymus nutans* by exogenous application of melatonin may involve ABA-dependent and ABA-independent pathways. *Sci. Rep.* **7**, 39865; doi: 10.1038/srep39865 (2017).

**Publisher's note:** Springer Nature remains neutral with regard to jurisdictional claims in published maps and institutional affiliations.

## Supplementary Material

Supplementary Dataset 1

Supplementary Information

## Figures and Tables

**Table 1:** Primer sequences used for qRT-PCR.

Primer Name	Primer Sequences (5′−3′)
*EnCBF9*-F	GGAGCTGCTTGTTCGACTAAT
*EnCBF9*-R	AACCGGGAACTCGACAGATA
*EnCBF14*-F	GAGGCTCTCACTATGAACGAAC
*EnCBF14*-R	TGGATGGTTTCTCTGTTTCTGT
*EnCOR14a*-F	CGATCAGCACGGAAGAAGAA
*EnCOR14a*-R	GCAAGTGCAAATTAGCTCATACA
*En18s rRNA-*F	GCCTCGTTTCCTGCTCTTAT
*En18s rRNA-*R	CCGCTCCAGAACAACATCT
