# Supplementary material for: Improved cold tolerance in Elymus nutans by exogenous application of melatonin may involve ABA-dependent and ABA-independent pathways
Source: Sci Rep. 2017 Jan 3;7:39865. doi: 10.1038/srep39865 (PMC5206618; doi:10.1038/srep39865)
Supplement: Supplementary Information [file srep39865-s2.pdf]

Improved cold tolerance in *Elymus nutans* by exogenous application of melatonin may involve ABA-dependent and ABA-independent pathways

Juanjuan Fu[1], Ye Wu[1], Yanjun Miao[2], Yamei Xu[2], Enhua Zhao[1], Jin Wang[1], Huaien Sun[1], Qian Liu[1], Yongwei Xue[3], Yuefei Xu[1,]*, Tianming Hu[1,]*

[1] Department of grassland science, College of Animal Science and Technology, Northwest A&F University, Yangling, Shaanxi, 712100, China

[2] College of Plant Science, Tibet Agriculture and Animal Husbandry College, Linzhi, Tibet, 860000, China

[3] Department of grassland ecology, College of Desertification Prevention Engineering, Ningxia Technical College of Wine and Desertification Prevention,Yongning, Yinchuan, 750001, China

Table S1. Effects of different melatonin concentrations on shoot length and fresh weight in DX and GN seedlings under control conditions.

	Shoot length (cm)		Fresh weight (g plant^{-1})	
	DX	GN	DX	GN
Control	30.2 ± 2.6	27.8 ± 2.4	0.52 ± 0.07	0.35 ± 0.03
1 uM MET	28.9 ± 3.1	28.5 ± 1.8	0.53 ± 0.04	0.40 ± 0.02
10 uM MET	29.5 ± 2.2	27.0 ± 2.1	0.48 ± 0.05	0.36 ± 0.05
50 uM MET	32.4 ± 2.5	29.5 ± 2.8	0.50 ± 0.03	0.33 ± 0.04
100 uM MET	31.2 ± 3.3	28.1 ± 3.0	0.51 ± 0.02	0.35 ± 0.02
300 uM MET	30.5 ± 2.8	27.2 ± 1.7	0.54 ± 0.04	0.36 ± 0.05

Each value rechayixianzhuxingbiaoji presents the mean of three replicates ± SD. An ANOVA test followed by Duncan's multiple range test ($P < 0.05$) was performed.

No significant difference marks indicate these data are not significantly difference at the 0.05 level.

Table S2. Effect of melatonin and ABA on electrolyte leakage, MDA content, H_2O_2 and superoxide radical accumulation in leaves of DX and GN under control conditions.

	Electrolyte leakage (%)		MDA content (nmol g^{-1} FW)		H_2O_2 content (μmol g^{-1} FW)		Superoxide radical content (nmol g^{-1} FW)	
	DX	GN	DX	GN	DX	GN	DX	GN
Control	7.52 ± 1.59	8.22 ± 1.23	2.05 ± 0.36	2.42 ± 0.21	0.29 ± 0.05	0.34 ± 0.06	0.41 ± 0.07	0.44 ± 0.05
MET	8.05 ± 1.43	8.12 ± 1.58	2.15 ± 0.48	2.53 ± 0.31	0.31 ± 0.03	0.42 ± 0.04	0.38 ± 0.04	0.48 ± 0.08
ABA	7.31 ± 1.51	7.56 ± 1.35	1.86 ± 0.21	2.01 ± 0.52	0.24 ± 0.02	0.51 ± 0.03	0.34 ± 0.05	0.51 ± 0.06
Fluridone	7.86 ± 1.63	8.35 ± 1.44	1.91 ± 0.28	2.38 ± 0.42	0.26 ± 0.03	0.38 ± 0.02	0.43 ± 0.03	0.41 ± 0.04
MET+F	7.16 ± 1.26	7.84 ± 1.66	2.03 ± 0.33	2.19 ± 0.29	0.33 ± 0.04	0.47 ± 0.05	0.31 ± 0.06	0.45 ± 0.05

Each value represents the mean of three replicates ± SD. An ANOVA test followed by Duncan's multiple range test ($P < 0.05$) was performed.

No significant difference marks indicate these data are not significantly difference at the 0.05 level.

Table S3. Effect of melatonin and ABA on non- enzymatic antioxidants GSH, AsA, total glutathione and ascorbate content in leaves of DX and GN under control conditions.

	GSH content (μg g^{-1} FW)		AsA content (μg g^{-1} FW)		Total glutathione content (μg g^{-1} FW)		Total ascorbate content (μg g^{-1} FW)	
	DX	GN	DX	GN	DX	GN	DX	GN
Control	85.25 ± 7.96	89.18 ± 8.56	17.56 ± 1.69	16.21 ± 1.89	165.12 ± 13.56	172.51 ± 12.85	36.25 ± 5.19	38.65 ± 4.02
MET	87.15 ± 9.36	88.58 ± 9.82	18.37 ± 2.12	15.43 ± 1.43	169.25 ± 11.83	181.36 ± 14.65	38..54 ± 4.56	40.82 ± 4.33
ABA	85.69 ± 9.85	90.23 ± 7.84	17.05 ± 1.81	16.83 ± 2.25	159.76 ± 12.18	176.26 ± 13.57	34.65 ± 5.13	41.06 ± 3.54
Fluridone	86.83 ± 8.05	87.95 ± 8.16	16.86 ± 2.05	15.61 ± 1.73	158.26 ± 11.51	178.82 ± 14.09	36.13 ± 4.28	37.86 ± 3.76
MET+F	84.66 ± 7.76	89.81 ± 9.15	18.16 ± 1.92	16.02 ± 1.81	162.38 ± 10.92	180.11 ± 12.67	37.15 ± 3.56	38.32 ± 4.24

Each value represents the mean of three replicates ± SD. An ANOVA test followed by Duncan's multiple range test ($P < 0.05$) was performed.

No significant difference marks indicate these data are not significantly difference at the 0.05 level.

Table S4. Effect of melatonin and ABA on the activities of antioxidant enzyme SOD, CAT, APX and GR in leaves of DX and GN under control conditions.

	SOD activity (U mg^{-1} Protein)		CAT activity (U mg^{-1} Protein)		APX activity (U mg^{-1} Protein)		GR activity (U mg^{-1} Protein)	
	DX	GN	DX	GN	DX	GN	DX	GN
Control	5.82 ± 1.85	5.13 ± 1.65	0.45 ± 0.08	0.41 ± 0.06	1.32 ± 0.19	1.21 ± 0.22	1.56 ± 0.17	1.71 ± 0.22
MET	6.15 ± 1.73	4.86 ± 1.58	0.55 ± 0.06	0.38 ± 0.04	1.49 ± 0.22	1.05 ± 0.18	1.65 ± 0.21	1.63 ± 0.17
ABA	5.64 ± 1.68	5.05 ± 1.46	0.42 ± 0.05	0.50 ± 0.05	1.26 ± 0.15	0.96 ± 0.17	1.39 ± 0.28	1.82 ± 0.18
Fluridone	5.37 ± 1.59	5.54 ± 1.32	0.49 ± 0.06	0.44 ± 0.03	1.42 ± 0.18	1.28 ± 0.28	1.52 ± 0.18	1.56 ± 0.20
MET+F	6.02 ± 1.43	4.95 ± 1.21	0.51 ± 0.07	0.38 ± 0.05	1.44 ± 0.21	1.15 ± 0.24	1.45 ± 0.23	1.65 ± 0.25

Each value represents the mean of three replicates ± SD. An ANOVA test followed by Duncan's multiple range test ($P < 0.05$) was performed.

No significant difference marks indicate these data are not significantly difference at the 0.05 level.

Table S5. Endogenous ABA concentration in leaves of DX and GN under control conditions.

**DX**	0 h	1 h	3 h	6 h	12 h	24 h	120 h
Control	12.00 ± 2.68	11.35 ± 2.19	12.65 ± 2.58	10.92 ± 3.16	11.65 ± 2.56	12.37 ± 2.69	11.53 ± 2.18
MET	13.21 ± 3.12	12.85 ± 2.35	13.05 ± 2.46	12.18 ± 2.54	11.48 ± 2.43	13.27 ± 3.11	13.08 ± 3.15
ABA	12.70 ± 2.54	13.25 ± 3.16	13.82 ± 2.64	12.31 ± 2.71	11.96 ± 2.68	13.07 ± 2.73	12.53 ± 2.81
Fluridone	13.05 ± 2.79	11.82 ± 2.85	13.15 ± 3.05	12.82 ± 2.38	13.52 ± 3.03	12.76 ± 2.81	13.08 ± 2.91
MET+F	12.25 ± 2.32	13.05 ± 3.36	13.52 ± 2.72	12.84 ± 2.62	13.18 ± 3.12	12.6 ± 3.02	13.37 ± 3.06

**GN**	0 h	1 h	3 h	6 h	12 h	24 h	120 h
Control	11.52 ± 2.65	10.56 ± 2.81	9.85 ± 2.35	12.05 ± 2.64	11.27 ± 2.16	10.87 ± 2.67	9.57 ± 2.35
MET	11.65 ± 2.16	12.19 ± 3.05	11.87 ± 2.54	13.05 ± 3.25	11.38 ± 2.03	12.36 ± 3.04	12.05 ± 2.16
ABA	10.48 ± 2.32	11.95 ± 2.56	10.86 ± 2.16	9.75 ± 2.58	10.16 ± 2.35	12.27 ± 2.81	9.92 ± 2.21
Fluridone	10.08 ± 3.04	9.56 ± 2.87	8.49 ± 2.21	9.16 ± 2.49	10.73 ± 2.61	9.36 ± 2.62	8.71 ± 2.51
MET+F	11.62 ± 2.83	13.18 ± 2.67	12.05 ± 2.94	11.85 ± 2.34	12.13 ± 2.73	11.91 ± 2.46	10.86 ± 2.73

ABA content (pmol g^{-1} FW).

Each value represents the mean of three replicates ± SD. An ANOVA test followed by Duncan's multiple range test ($P < 0.05$) was performed.

No significant difference marks indicate these data are not significantly difference at the 0.05 level.

Table S6. Expression of *EnCBF9, EnCBF14, EnCOR14a* genes identified in our previous transcriptome data (accession No: SRP074469) in leaves of DX and GN under cold stress.

**DX**	0 h	3 h	24 h	120 h
EnCBF9	0.4433	11.7167	0.6633	0.5467
EnCBF4	0.0367	5.8433	0.6233	0.3167
EnCOR14a	4.0267	64.8133	1042.6633	1035.7067

**GN**	0 h	3 h	24 h	120 h
EnCBF9	0.2733	12.5333	0.3233	0.1733
EnCBF4	0.01	10.6833	0.12	0.19
EnCOR14a	6.1333	98.4733	844.48	652.9367

Gene expression levels were calculated by the Fragments Per Kb per Million fragments (FPKM).

DX

**Gene name**	**Unigene ID**	**SwissProt annotation**	**GO annotation**
EnCBF9	Unigene86386_All	CRT/DRE binding factor 9 [Triticum aestivum]	F: DNA binding; F: sequence-specific DNA binding; F: transcription factor activity; C: nucleus; P: regulation of transcription, DNA-templated
EnCBF14	Unigene13886_All	CRT/DRE binding factor 14 [Triticum monococcum]	F: DNA binding; F: sequence-specific DNA binding transcription factor activity; C: nucleus; P: regulation of transcription, DNA-templated
EnCOR14a	Unigene54212_All	Cold-responsive protein COR14a [Aegilops tauschii]	P: response to water; P: response to stress

GN

**Gene name**	**Unigene ID**	**SwissProt annotation**	**GO annotation**
EnCBF9	Unigene6166_All	CBF9 [Hordeum vulgare]	F: DNA binding; F: sequence-specific DNA binding transcription factor activity; C: nucleus; P: regulation of transcription, DNA-templated
EnCBF14	Unigene24735_All	CBF14 [Hordeum vulgare subsp. vulgare]	F: DNA binding; F: sequence-specific DNA binding transcription factor activity; C: nucleus; P: regulation of transcription, DNA-templated
EnCOR14a	CL3945.Contig2_All	Cold-responsive protein COR14a [Aegilops tauschii]	P: response to water; P: response to stress
